# Multiple nodules covering the forearm: a case of fish-sting granuloma^☆^

**DOI:** 10.1016/j.abd.2022.06.012

**Published:** 2023-12-14

**Authors:** Teng Chao Wei, Xian Mei Lu, Fang Fang Bao, Hong Liu

**Affiliations:** Shandong Provincial Hospital for Skin Diseases & Shandong Provincial Institute of Dermatology and Venereology, Shandong First Medical University & Shandong Academy of Medical Sciences, Jinan, Shandong, China

Dear Editor,

*Mycobacterium marinum* is one of the non-tuberculous mycobacteria that most often causes skin and soft tissue infections in patients, especially those exposed to aquatic environments or marine life, hence named pool granuloma and fish tank granuloma.^1^ With the increased consumption of seafood, *Mycobacterium marinum* infection due to fish sting injuries is on the rise. Here, we report a postoperative breast cancer patient with local lymphatic reflux insufficiency who developed diffuse nodules on the right upper extremity after an accident, while handling fish.

A 78-year-old woman presented with diffuse swelling erythematous nodules and crusted plaques on her right upper extremity for eight weeks. The patient had a broken wound on her right index finger which was caused by fish handling. The patient underwent a right mastectomy and lymphatic dissection for breast cancer 12 years ago. The physical examination showed a wound on the right index finger and diffused nodules of the right upper extremity (Fig. 1). Blood test results were unremarkable. Histopathological examination showed epidermal hyperkeratosis, and intra-dermal epithelioid cell granuloma with lymphocytes, Ziehl-Neelsen staining reveals acid-fast bacillus. T-SPOT.TB was double-positive, and tissue culture suggested *Mycobacterium marinum* infection (Fig. 2), so the diagnosis of *Mycobacterium marinum* infection was made. After six months of combined oral rifampin (0.6 g/d) and clarithromycin (0.5 g/Bid) treatment, all nodules subsided, leaving scars.

**Figure 1:**
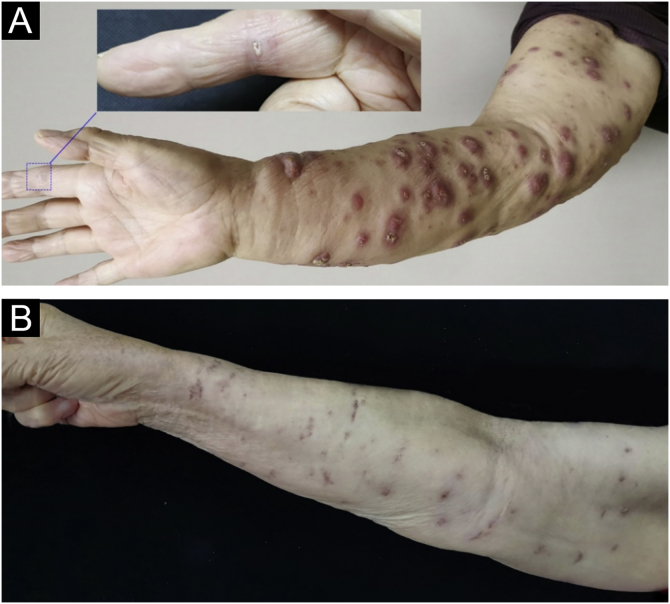
(A) The finger was injured while handling the fish and diffuse nodules of the right upper extremity (At the time of the visit). (B) After six months of combined oral rifampin and clarithromycin treatment, the nodules all subsided, leaving scars.

**Figure 2:**
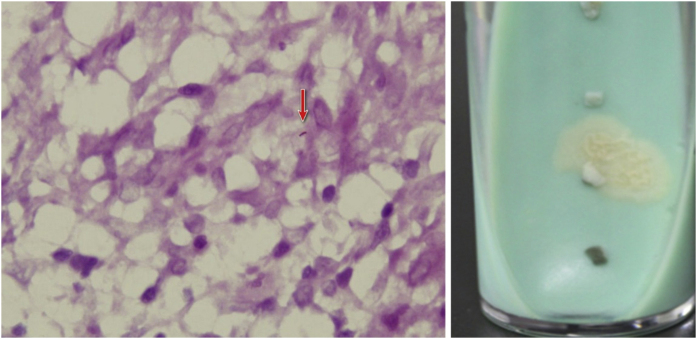
(A) The Ziehl-Neelsen stain showed an acid-fast bacillus, red arrow indicates acid-fast bacillus. (B) Smooth, yellow colonies visible on Löwenstein-Jensen medium after 14 days culture.

Marine mycobacterium is widely distributed in water environments and used to be common among swimmers in swimming pools and workers in fishing grounds. In China, due to eating habits, cooks and housewives are vulnerable to infection when handling fish. *Mycobacterium marinum* was originally isolated from seabass. Fish infected with *Mycobacterium marinum* have been reported as sturgeon, seabass and so on.^2^ This case was caused by an accident with seabass. In the past decade, outbreaks of *Mycobacterium marinum* in seabass have been reported in the United States.^3^

The typical clinical presentation is the formation of nodular or sporadic filamentous limb lesions, manifesting as deeper interstitial infections such as tenosynovitis and osteomyelitis.^2^ Extensive dissemination may occur in a minimal number of immunocompromised hosts.^4^ Previously reported cases of widespread dissemination have been associated with immunosuppression as well as the presentation with ulcers, skin nodules, and nodular lymphangitis. In the present case, there was lymphatic reflux disorder after the right mastectomy, so the patient developed widespread skin lesions along the right upper extremity after infection. Culture is still the gold standard for the diagnosis of this infection. For treatment, we used combined oral rifampin (0.6 g/d) and clarithromycin (0.5 g/bid), and the patient was cured after 6 months of treatment.

In summary, we report a case of infection of *Mycobacterium marinum* infection which occurred in postoperative patients with breast cancer and showed spread distribution on the upper limb. Clinicians should be alert to these diseases and give enough antibiotics for a full course of treatment. Recognizing the variable clinical presentation can help in early diagnosis and determine prognosis.

## Financial support

None declared.

## Authors’ contributions

TengChao Wei: Approval of the final version of the manuscript; critical literature review; analysis and interpretation; preparation and writing of the manuscript.

XianMei Lu: Pathological examinations; analysis and interpretation.

FangFang Bao: Performed laboratory examinations; analysis and interpretation.

Hong Liu: Critically reviewed the manuscript.

## Conflicts of interest

None declared.
